# Prognostic values of clinical and molecular features in HER2 low-breast cancer with hormonal receptor overexpression: features of HER2-low breast cancer

**DOI:** 10.1007/s12282-022-01364-y

**Published:** 2022-06-21

**Authors:** Mengdi Chen, Weilin Chen, Deyue Liu, Weiguo Chen, Kunwei Shen, Jiayi Wu, Li Zhu

**Affiliations:** 1 Department of General Surgery, Comprehensive Breast Health Center, Ruijin Hospital, Shanghai Jiao Tong University School of Medicine, 197 Ruijin Er Road, Huangpu District, Shanghai, 200025 China; 2 Department of Thyroid and Breast Surgery, Shanghai General Hospital, Shanghai Jiao Tong University School of Medicine, 85 Wujin Road, Hongkou District, Shanghai, 200080 China

**Keywords:** Breast cancer, HER2-low, Hormone receptor-positive, Prognosis, Recurrence score

## Abstract

**Background:**

Human epidermal growth factor receptor 2 (HER2) low breast cancer was considered as a distinct subtype different from HER2-zero breast cancer. Our study aimed to investigate the prognostic values of clinicopathological features and recurrence score (RS) in HER2-low and HER2-zero hormone receptor (HR)-positive breast cancer patients.

**Methods:**

A total of 2099 HR + primary female breast cancer patients diagnosed between Jan 2009 and Jan 2019 were collected. Tumors with immunohistochemistry 1 + /2 + and negative in situ hybridization results were defined as HER2-low. We compared the clinical and genetical features of HER2-low (*n* = 1732) and HER2-zero (*n* = 367) breast cancer and their prognostic values.

**Results:**

Estrogen receptor (ER) high expression (> 90%) was more common in HER2-low breast cancer than HER2-zero breast cancer (78.2% vs 58.6%, *p* < 0.01). Five-year disease-free survival (DFS) was similar between HER2-zero and HER2-low subgroups (92.3% vs 93.3%, *p* = 0.83). The predictive value of RS was only significant in HER2-zero patients (*p* = 0.03). The proliferation-related genes performed well in predicting DFS in HER2-zero patients, but not in HER2-low patients (*p* for interaction < 0.01). The higher HER2 module score was correlated with worse DFS only in HER2-low patients (*p* = 0.04).

**Conclusion:**

We observed similar survival outcomes between HER2-low and HER2-zero HR + patients. HER2-low patients had a higher proportion of ER high expressed tumors than HER2-zero patients did. RS and its proliferation module might be less clinically meaningful to HER2-low patients.

**Supplementary Information:**

The online version contains supplementary material available at 10.1007/s12282-022-01364-y.

## Introduction

Human epidermal growth factor receptor 2 (HER2) was one of the most important biomarkers in breast cancer. Based on the immunohistochemistry (IHC) and in situ hybridization (ISH) results, tumors were previously defined as HER2-positive and HER2-negative. Only HER2-positive patients could benefit from traditional anti-HER2 agents [1–3]. Recently, the binary classification was challenged and oncologists paid more attention to tumor cells with HER2 low expression but negative ISH status. They were re-classified into a distinct new subtype: HER2-low breast cancer [4].

HER2-low subtype constitutes 45–55% of invasive breast cancers [5]. Previous studies were insufficient for clinicians to differentiate them from HER2-zero subtype [6–10]. They were treated as luminal-like or triple-negative subtype according to the hormone receptor (HR) status. Approximately 50–80% of traditional HER2 non-amplified breast cancers were HR-positive [8, 11]. What’s more important, recent analysis suggested that the difference between HER2 low expression and HER2 IHC 0 had stronger clinical influence in HR-positive breast cancer than in triple-negative breast cancer [11]. The 21-gene recurrence score (RS) [12] is the most widely used assay, providing more accurate prognosis information. The ranges of RS allow certain group of patients, including HER2-low patients, to avoid chemotherapy. Two HER2 amplification-related genes, growth factor receptor-bound protein 7 (GRB7) and HER2, were important components of RS, and they might play different roles in HER2-low and HER2-zero tumors. However, whether low HER2 expression would influence the predictive value of RS has never been investigated before.

Therefore, in our study, we aim to explore the traditional features and RS (including the constituent genes) in HER2-low and HER2-zero patients and evaluate their prognostic roles.

## Patients and methods

### Patients

In total, clinical data of 2099 HR-positive primary female breast cancer patients diagnosed between Jan 2009 and Jan 2019 were retrospectively collected from Shanghai Jiao Tong University Breast Cancer Data Base (SJTU-BCDB). All histological and IHC tumor slides were evaluated by two pathologists with a light microscope at magnification of × 100. Inclusion criteria were listed as follows: (1) estrogen receptor (ER) positivity, defined as ≥ 1% immuno-reactive tumor cell nuclei determined by IHC staining [13]; (2) detailed HER2 IHC and ISH results; (3) intact 21-gene report; (4) non-metastatic. The median follow-up time was 50.19 (range 2.54–120.00) months.

### HER2 status

HER2-zero was defined as IHC 0. HER2-low was defined as IHC 1 + /2 + and negative ISH result. According to American Society of Clinical Oncology (ASCO) guidelines [14], HER2 non-amplification would be determined when the ratio of HER2/chromosome 17 centromere was ≥ 2.2 (before 2013) or ≥ 2.0 (after 2013), or HER2 Copy Number was ≥ 6.0.

### The 21-gene assay

The 21-gene tests were performed on formalin-fixed, paraffin-embedded samples [15]. According to the guidelines in Ruijin Hospital, RNA was extracted and purified using the RNeasy FFPE kit (QIAGEN, Hilden, Germany). Total RNA content was quantified after confirmed the absence of DNA contamination. Gene-specific reverse transcription was conducted using Omniscript RT kit (Qiagen, 205111, Germany) followed by standardized quantitative reverse transcriptase polymerase chain reactions (RT-PCR) in 96-well plates with Applied Biosystems (Foster City, CA) 7500 Real-Time PCR system. Expression of each gene was measured in triplicate, and normalized relative to a set of five reference genes. The RS thresholds were set to 18 and 30 before Dec 2015 [12] and 11 and 25 after Jan 2016 [16] according to the publication date of the TAILORx trial and the change of clinicians’ choices in Ruijin Hospital.

### Statistical analysis

Disease-free survival (DFS) was defined as the period from the time of first treatment for breast cancer to the time of first event, including local, regional or distant recurrence, contralateral breast cancer, secondary malignancy or death as a result of any cause. The Kaplan–Meier survival analysis and univariate and multivariate Cox regression models were performed respectively in patients according to different genetic risks and HER2 status. All the tests were performed on the R Studio version 1.2.5019 based on R version 4.0.3.

## Results

### Baseline characteristics of HER2-low and HER2-zero patients

Among 2099 HR-positive cases, 82.5% were HER2-low (*n* = 1732%). Eight hundred and seventy eight cases were HER2 1 + and 854 were HER2 2 + . We did not find significant difference when comparing the age, menopause status, histological grade, progesterone receptor (PR) status, American Joint Committee on Cancer (AJCC) stage of HER2-low and HER2-zero subgroups (Table 1). Sixteen percent of HER2-low patients and 10.5% of HER2-zero patients (*p* = 0.01) had specific pathologic types beyond invasive ductal and lobular cancer. HER2-low patients also had a higher proportion of tumors with high histologic grades (*p* = 0.02). We divided ER IHC expression according to its median percentage: < 90% and ≥ 90%. ER high expression was more common in HER2-low tumors than in HER2-zero tumors (78.2% vs 58.6%, *p* < 0.01).

**Table 1:** Basic Characteristics of 2,099 HR + breast cancer patients from SJTU-BCDB

Characteristics	HER2 -zero *n* = 367	HER2-low *n* = 1732	*p* value
Age			0.39
≤ 50	110 (30.0)	562 (32.4)	
> 50	257 (70.0)	1,170 (67.6)	
Menopause status			0.61
Premenopausal	123 (33.5)	607 (35.0)	
Postmenopausal	244 (66.5)	1,125 (65.0)	
Pathology			0.01
IDC	294 (80.1)	1,478 (85.3)	
ILC	14 (3.8)	72 (4.2)	
others	59 (16.1)	182 (10.5)	
Grade			0.02
I	36 (9.8)	147 (8.5)	
II	173 (47.1)	1,027 (59.3)	
III	77 (21.0)	312 (18.0)	
Unknown	81 (22.1)	246 (14.2)	
ER positivity (%)			< 0.01
< 90	152 (41.4)	377 (21.8)	
≥ 90	215 (58.6)	1355 (78.2)	
PR			0.06
Positive	313 (85.3)	1,540 (88.9)	
Negative	54 (14.7)	192 (11.1)	
Ki-67 (%)			0.30
< 20	213 (58.0)	951 (54.9)	
≥ 20	154 (42.0)	781 (45.1)	
pT			0.68
1	262 (71.4)	1,215 (70.2)	
2–4	105 (28.6)	517 (29.8)	
pN			0.47
0	313 (85.3)	1,448 (83.6)	
1–2	54 (14.7)	284 (16.4)	

*IDC*, invasive ductal carcinoma; *ILC*, invasive lobular carcinoma; *ER*, estrogen receptor; *PR*, progesterone receptor

We found no difference in the distribution of genetic risk and postoperative treatment between HER2-low and HER2-zero patients, regardless of the date of diagnosis (Table 2).

**Table 2:** RS range and treatment after surgery of patients diagnosed in different periods

Characteristics	HER2-zero	HER2-low	*p* value
*Before Dec 2015*			
RS			0.36
< 18	48	192	
18–30	70	374	
> 30	55	275	
Endocrine therapy			0.39
AI (± OFS)	104	499	
SERM (± OFS)	68	341	
Others	1	1	
Chemotherapy			0.22
No	95	418	
Yes	77	422	
Radiotherapy			0.72
No	101	478	
Yes	71	362	
*After Jan 2016*			
RS			0.46
< 11	7	25	
11–25	96	483	
> 25	91	383	
Endocrine therapy			0.43
AI (± OFS)	142	615	
SERM (± OFS)	52	275	
Others	0	1	
Chemotherapy			0.57
No	101	441	
Yes	93	450	
Radiotherapy			0.71
No	96	425	
Yes	98	466	

*RS*, recurrence score; *AI*, aromatase inhibitor; *SERM*, selective estrogen receptor modulator; *OFS*, ovarian function suppression

**Fig. 1:**
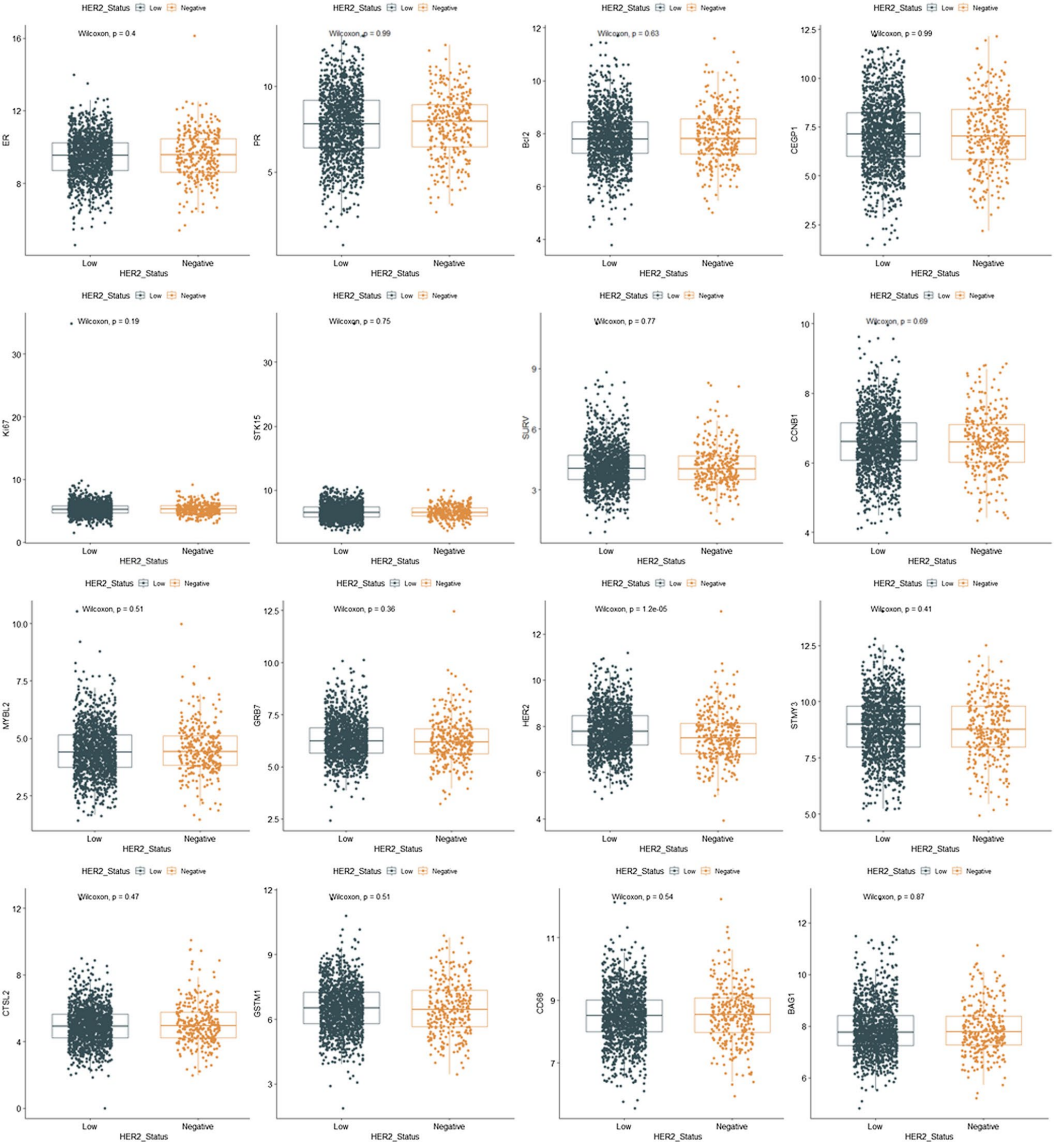
The expression of RS genes in HER2-low and HER2-zero patients

We also analyzed the expression of RS genes in HER2-low and HER2-zero groups, most of which were similar. Only the level of HER2 mRNA was slightly higher in HER2-low subgroup than in HER2-zero cohort (Fig. 1). HER2 and ER mRNA levels were positively correlated in both HER2-zero and HER2-low patients (Supplemental Fig. 1).

### The survival outcomes of HER2-low and HER2-zero patients

**Fig. 2:**
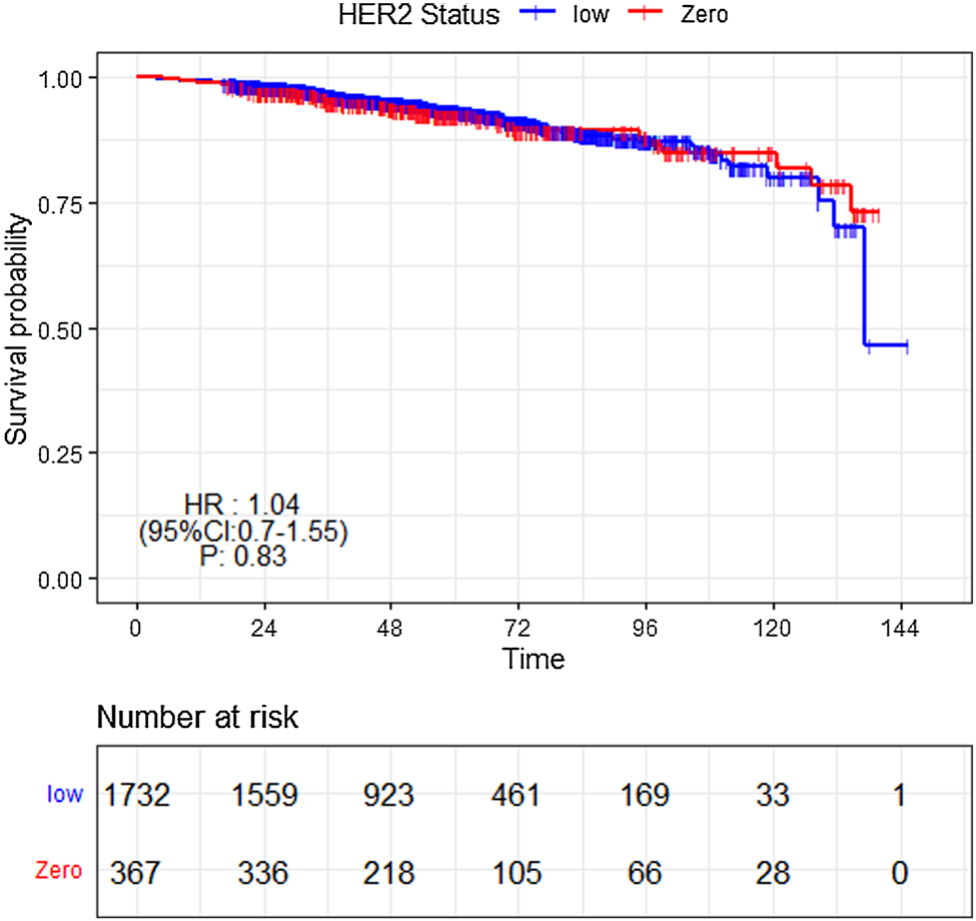
Association between HER2 status and DFS in HR + breast cancer patients

A total of 146 DFS events occurred during the follow-up period (33 in HER2-zero and 123 in HER2-low subgroups). The survival outcomes (Fig. 2) had no significant difference between HER2-low and HER2-zero subgroups (hazard ratio 1.04, 95% CI 0.7–1.55, *p* = 0.8). In subgroup analysis, the survival outcomes of HER2-low and HER2-zero patients remained similar regardless of whether they received OFS or chemotherapy (Supplemental Fig. 2). We investigated the impacts of clinicopathological factors on DFS in subgroups with different HER2 status (Table 3). In HER2-zero cohort, PR positivity (hazard ratio = 0.45 95% CI 0.21–0.97, *p* = 0.04) predicted better survival and higher pN stage (hazard ratio = 2.63, 95% CI 1.09–6.34, *p* = 0.03) predicted worse survival after adjustment. In HER2-low cohort, only high Ki-67 index was associated with worse survival (hazard ratio = 1.57, 95% CI 1.02–2.44, *p* = 0.04) in the multivariate analysis.

**Table 3:** The impact of clinicopathological factors on DFS in HER2-low and HER2-zero patients

Factors	Univariate analysis	*p* value	Multivariate analysis	*p* value
*HER2-zero*				
Age (years)				
> 50 vs ≤ 50	1.08 (0.51–2.66)	0.85		
Menopause				
Post vs Pre	1.37 (0.65–2.88)	0.42		
Grade				
III vs I–II	1.67 (0.76–3.65)	0.20		
ER positivity (%)				
≥ 90 vs < 90	0.46 (0.21–1.02)	0.06	0.45 (0.20–1.02)	0.06
PR status				
Pos vs Neg	0.39 (0.18–0.81)	0.01	0.45 (0.21–0.97)	0.04
Ki-67 (%)				
≥ 20 vs < 20	1.68 (0.82–3.43)	0.16		
pT				
2–4 vs 1	1.99 (0.98–4.01)	< 0.01	1.61 (0.76–3.44)	0.22
pN				
≥ 1 vs 0	2.84 (1.24–6.49)	0.01	2.63 (1.09–6.34)	0.03
*HER2-low*				
Age (years)				
> 50 vs ≤ 50	0.90 (0.62–1.31)	0.60		
Menopause				
Post vs Pre	0.91 (0.63–1.30)	0.60		
Grade				
III vs I–II	1.73 (1.15–0.62)	0.01	1.32 (0.84–2.08)	0.23
ER positivity (%)				
≥ 90 vs < 90	0.66 (0.45–0.98)	0.04	0.69 (0.45–1.06)	0.09
PR status				
Pos vs Neg	0.76 (0.47–1.24)	0.27		
Ki-67 (%)				
≥ 20 vs < 20	1.71 (1.20–2.45)	< 0.01	1.57 (1.02–2.44)	0.04
pT				
2–4 vs 1	2.19 (1.54–3.13)	< 0.01	1.43 (0.95–2.16)	0.09
pN				
≥ 1 vs 0	1.15 (0.70–1.91)	0.58		

*ER*, estrogen receptor; *PR*, progesterone receptor; *HER2*, human epidermal growth factor receptor 2

### The predictive value of RS between different HER2 status

**Fig. 3:**
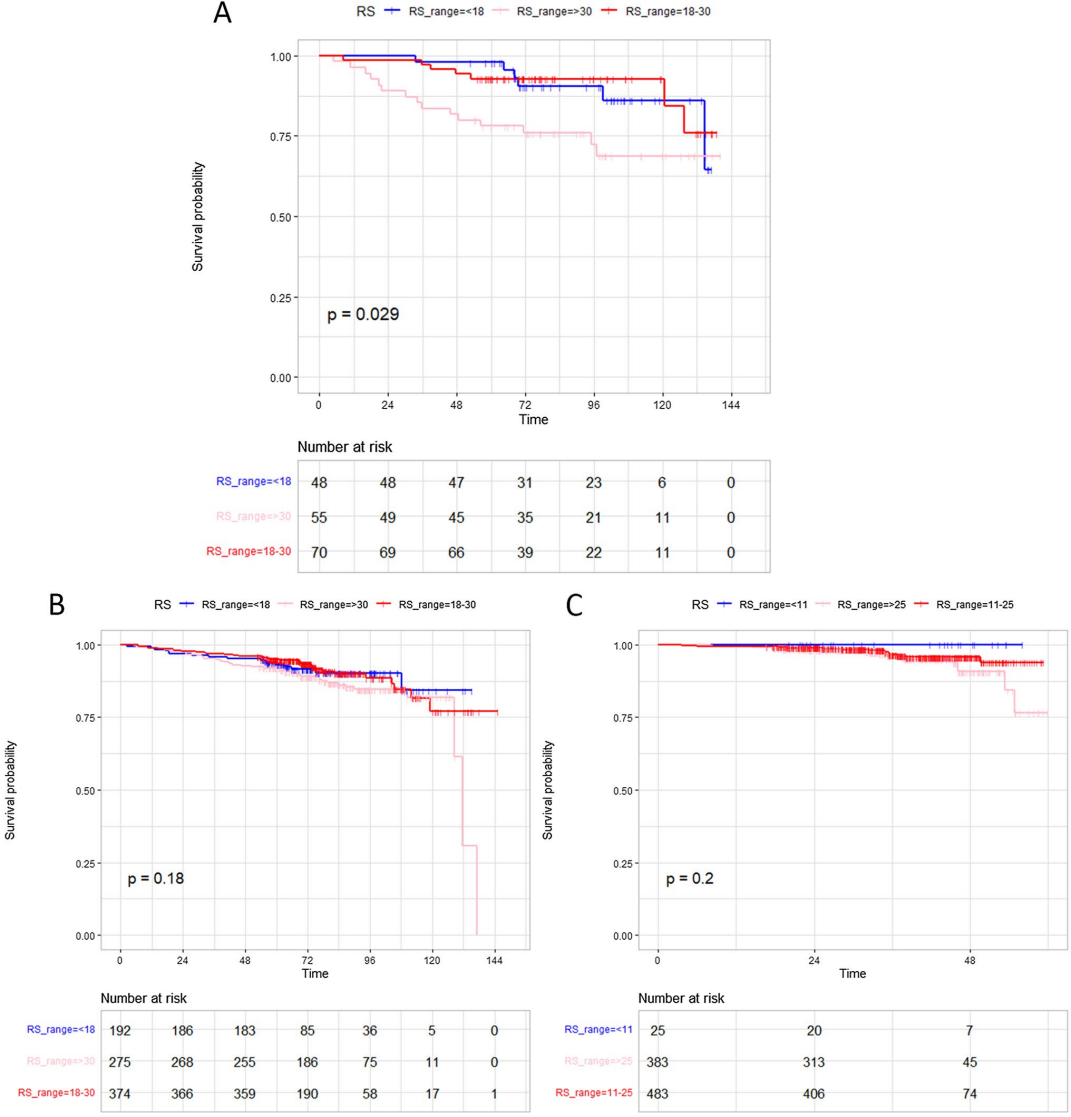
DFS of patients with different RS. **A** HER2-zero, before Dec 2015; **B** HER2-low patients, before Dec 2015; **C** HER2-low, after Dec 2015

**Fig. 4:**
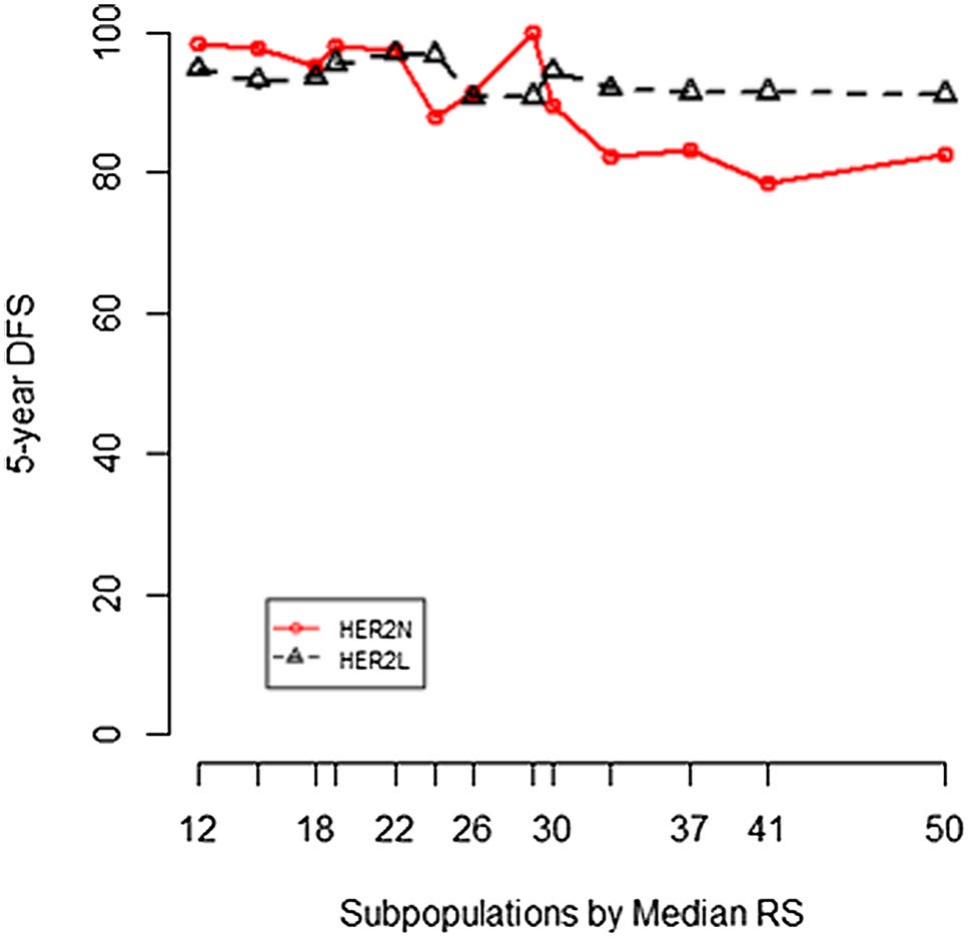
Five-year DFS of patients with different HER2 status by median RS

We tested the predictive value of RS in three cohorts: HER2-zero patients diagnosed before Dec 2015, HER2-low before Dec 2015, and HER2-low after Jan 2016 (Fig. 3). In HER2-zero cohort, patients with high RS showed significant worse survival than those with low/intermediate RS (Fig. 3A, *p* = 0.03). However, in HER2-low patients, RS was not significantly correlated with the survival outcome (Fig. 3B, C). The analysis in HER2-zero patients after Jan 2016 (Supplemental Fig. 3) showed no significant difference probably due to limited DFS events (*n* = 5). We also analyzed the impact of continuous RS on survival. The subpopulation treatment effect pattern plot (STEPP) analysis [17] showed that when RS was over 30, HER2-low patients had a better DFS than HER2-zero ones did (Fig. 4). About 70% of patients with high genetic risk (RS > 30 before Dec 2015 and RS > 25 after Jan 2015) received chemotherapy. To ensure that the survival analysis was not affected by disproportionate treatment, we compared the postoperative chemotherapy administration between HER2-low and HER2-zero patients with different genetic risks. The proportion of HER2-low patients received chemotherapy was similar to that of HER2-zero patients (Supplement Table 1).

### The impact of RS modules on DFS according to HER2 status

We further evaluated the prognostic roles of the constituent modules and genes of RS in patients with different HER2 status (Table 4).

**Table 4:** The impact of RS modules and genes on DFS according to HER2 status

Factors	HER2-zero	*p* value	HER2-low	*p* value	*p*_interaction_
ER Module					
ER	1.20 (0.88–1.65)	0.25	1.01 (0.85–1.19)	0.95	0.36
PgR	0.84 (0.67–1.06)	0.15	0.97 (0.87–1.08)	0.55	0.30
Bcl2	0.88 (0.58–1.32)	0.53	0.87 (0.71–1.06)	0.16	0.67
SCUBE2	0.89 (0.70–1.13)	0.34	0.97 (0.86–1.09)	0.56	0.39
ER module	0.84 (0.58–1.21)	0.34	0.92 (0.76–1.11)	0.38	0.52
Proliferation module					
Ki-67	2.20 (1.46–3.30)	< 0.01	1.02 (0.88–1.19)	0.80	< 0.01
STK15	1.85 (1.26–2.72)	< 0.01	1.02 (0.89–1.17)	0.78	< 0.01
Survivin	1.72 (1.25–2.37)	< 0.01	1.06 (0.89–1.26)	0.53	0.03
CCNB1	1.48 (0.90–2.44)	0.12	1.19 (0.95–1.50)	0.14	0.64
MYBL2	1.52 (1.15–2.02)	< 0.01	1.08 (0.90–1.28)	0.41	0.12
Prol module	7.22 (2.49–20.93)	< 0.01	0.81 (0.40–1.66)	0.57	< 0.01
HER2 module					
GRB7	1.39 (1.04–1.87)	0.03	1.01 (0.82–1.24)	0.92	0.27
HER2	1.02 (0.70–1.49)	0.91	0.99 (0.81–1.23)	0.98	0.76
HER2 module	1.32 (0.71–2.48)	0.38	1.89 (1.02–3.48)	0.04	0.32
Invasion module					
MMP11	1.11 (0.83–1.48)	0.49	1.20 (1.04–1.39)	0.02	0.53
CTSL2	1.39 (1.06–1.83)	0.02	1.08 (0.90–1.29)	0.42	0.30
Inv module	1.58 (1.04–2.38)	0.03	1.30 (1.05–1.61)	0.02	0.68
GSTM1	1.15 (0.82–1.62)	0.43	0.85 (0.70–1.02)	0.08	0.32
CD68	1.57 (1.02–2.41)	0.04	1.02 (0.80–1.30)	0.87	0.20
BAG1	1.13 (0.73–1.77)	0.58	1.01 (0.82–1.24)	0.96	0.99

*RS*, recurrence score; *HER2*, human epidermal growth factor receptor 2; *DFS*, disease-free survival

In HER2-zero subgroup, the proliferation module and most of its constituent genes were negatively associated with DFS. Although high GRB7 expression was a possible predictor of poorer survival (hazard ratio = 1.39, 95%CI 1.04–1.87, *p* = 0.03), the HER2 module did not have significant prognostic value. The high invasion module score was associated with worse survival. No significant correlation was found between the ER module and DFS.

In HER2-low subgroup, we found that the proliferation-related genes could not predict the survival outcome. Meanwhile, the higher HER2 module score was associated with worse DFS (hazard ratio = 1.89, 95% CI 1.02–3.48, *p* = 0.04), which was not observed in HER2-zero patients. The invasion module remained a negative prognostic factor. The impact of ER module on DFS was not obvious.

Further, the interaction test showed significant correlation between most proliferation-related genes and HER2 status. Similar results were found in multivariate analysis adjusted by AJCC T stage and N stage, which was presented in Supplemental Table 2. The interaction test results implied that the correlation between the HER2 module (or the invasion module) and HER2 status was not significant.

## Discussion

Recently, the outstanding therapeutic effects of novel antibody–drug conjugates (ADCs) [18, 19] on HER2-low breast cancer had arouse the interest in this potential new subtype. Our study compared the clinicopathological characteristics and the RS gene expression between HER2-low and HER2-zero HR-positive early breast cancer patients. We found that HER2 low expression had a positive correlation with ER expression. RS and the proliferation module performed better in predicting DFS of HER2-zero patients than HER2-low patients. Our results provided important information about the usage of RS in HER2-low patients.

The distributions of most clinicopathological features and gene expression were similar in HER2-low and HER2-zero patients. However, the ER protein of HER2-low breast cancer had more chance to be highly expressed compared with HER2-zero tumors. The expression data derived from RS reports also showed an important correlation between HER2 and ER mRNA levels. This was in accordance with previous evidence in HER2-negative patients [20]. Several studies reported that a bidirectional cross-talk existed between ER and HER2 [21], which might disturb the endocrine therapy [22]. Whether the close correlation between ER and HER2 in HER2-low/HR + breast cancer patients would influence the treatment effects of endocrine drugs should be further investigated.

The survival analysis did not show any significant difference between HER2-low and HER2-zero patients. Although previous studies demonstrated that moderate HER2 expression might be an unfavorable prognostic factor [6, 23], our study showed that HER2 status (low/zero) was not an independent prognostic factor at least in HR-positive patients. Two recent studies also found HER2-low and HER2-zero HR + breast cancer patients had similar survival outcomes [7, 8]. Furthermore, one research demonstrated that instead of low HER2 expression [8], it was hormone receptor and its related genes that might be the actually dominated oncological drivers for HER2-low/HR + breast cancer [7].

We further evaluated the prognostic value of the RS modules in HER2-low and HER2-zero patients. Of note, almost all the proliferation-related genes were strong unfavorable prognostic factors in HER2-zero patients. However, they lost predictive value in HER2-low patients. Interestingly, protein expression of Ki-67 showed prognostic value in HER2-low patients, while mRNA level of Ki-67 did not. The results difficult to reproduce and lack of consensus on Ki-67 threshold might undermine the reliability of the prognostic value of protein expression of Ki-67 [24–26]. Therefore, proliferation-related multi-genes might reflect the proliferation ability of tumors better than protein expression of Ki-67 alone. The interaction test confirmed that the proliferation module performed differently when HER2 status changed. An implication was that RS might amplify the roles of proliferation-related genes in HER2-low breast cancer. Inconsistency also existed in impacts of the HER2 module on DFS between two subgroups. The high HER2 module score was associated with poor DFS in HER2-low population. In HER2-zero cohort, the HER2 module did not show important impact on DFS.

We also conducted survival analysis respectively in HER2-zero and HER2-low patients to test whether RS would maintain its prognostic value regardless of HER2 status. Since the acknowledged RS thresholds had changed in Ruijin Hospital after the publishing of the TAILORx study, we used different thresholds according to the date of diagnosis. Interestingly, we observed that RS had good performance as a DFS predictor in HER2-zero patients but not in HER2-low patients. The STEPP analysis showed an obvious survival discrepancy between HER2-low and HER2-zero patients when RS was > 30. According to the RS algorithm [12], the coefficient of the proliferation module was the highest among four modules. The absence of its predictability might affect the performance of RS in HER2-low breast cancer. Therefore, we hypothesized that RS was more applicable to HER2-zero patients and a refined RS range was needed in HER2-low patients.

Our study had several limitations. First, we conducted our study based on the retrospective data; thus, potential bias was unavoidable. For instance, some patients would be excluded because of incomplete record. The proportion of HER2 low cases in our study was larger than that was reported in previous studies (approximately 55–65%), probably because we only included patients with 21-gene test reports. Second, the follow-up period of our patients might be not long enough for HR + breast cancer. Therefore, we could not investigate the performance of RS in predicting DFS of HER2-zero patients diagnosed after Jan 2016 due to limited DFS events. Nevertheless, the result based on patients between 2009 and 2015 proved the predictive value of RS.

In conclusion, our study found that HER2 low expression might not be a prognostic factor in HR + patients. HER2-low patients had a higher proportion of ER high expressed tumors than HER2-zero one did. RS and its proliferation module might be less applicable to HER2-low patients. Further research should focus on the refinement of RS range in HER2-low HR-positive breast cancer.

## Supplementary Information

Below is the link to the electronic supplementary material.

Supplemental Figure 1 Correlation between HER2 and ER expression in HER2-zero and HER2-low HR+ breast cancer patients (TIF 2354 kb)

Supplemental Figure 2 Subgroup analysis of DFS in HER2-low and HER2-zero patients. (A) with chemotherapy; (B) without chemotherapy; (C) with OFS; (D) without OFS (JPG 708 kb)

Supplemental Figure 3 DFS of HER2-zero patients diagnosed after Dec 2015 (PNG 18 kb)

Supplementary file4 (DOCX 21 kb)

## Data Availability

The data used to support the findings of this study are available from the corresponding author upon request.
