# Supplementary figures and images for: Prognostic values of clinical and molecular features in HER2 low-breast cancer with hormonal receptor overexpression: features of HER2-low breast cancer
Source: Breast Cancer. 2022 Jun 21;29(5):844–53. doi: 10.1007/s12282-022-01364-y (PMC9385837; doi:10.1007/s12282-022-01364-y)

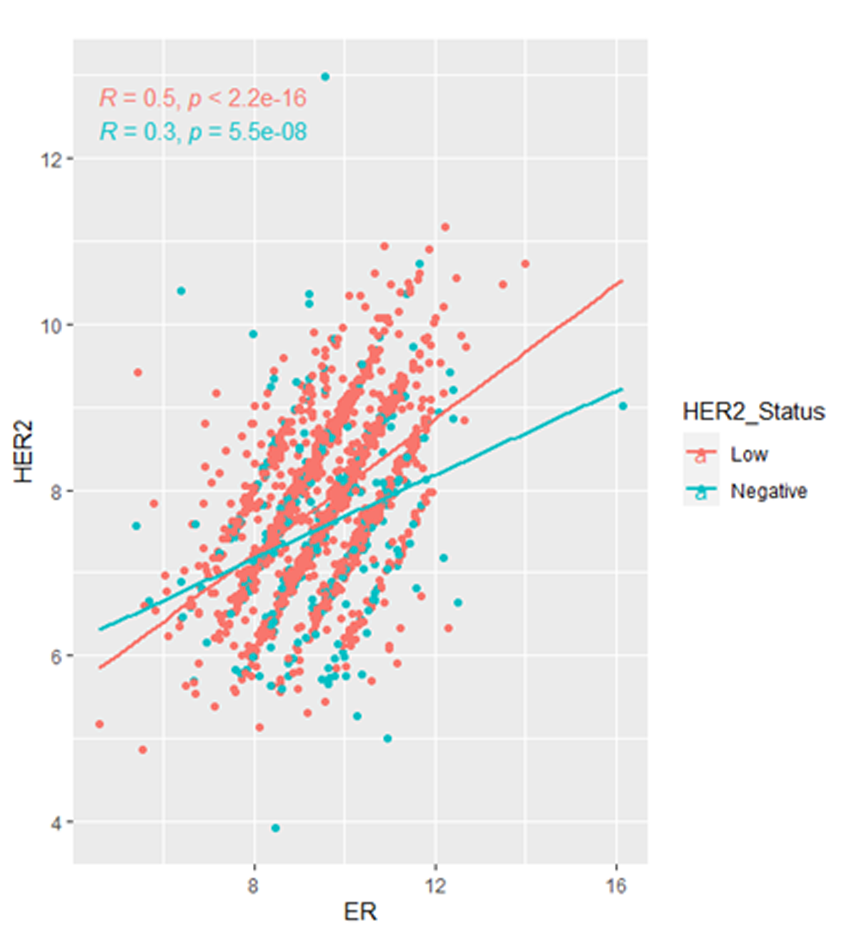

Supplement: Supplementary file 1 — Supplemental Figure 1 Correlation between HER2 and ER expression in HER2-zero and HER2-low HR+ breast cancer patients (TIF 2354 kb) [file 12282_2022_1364_MOESM1_ESM.tif]

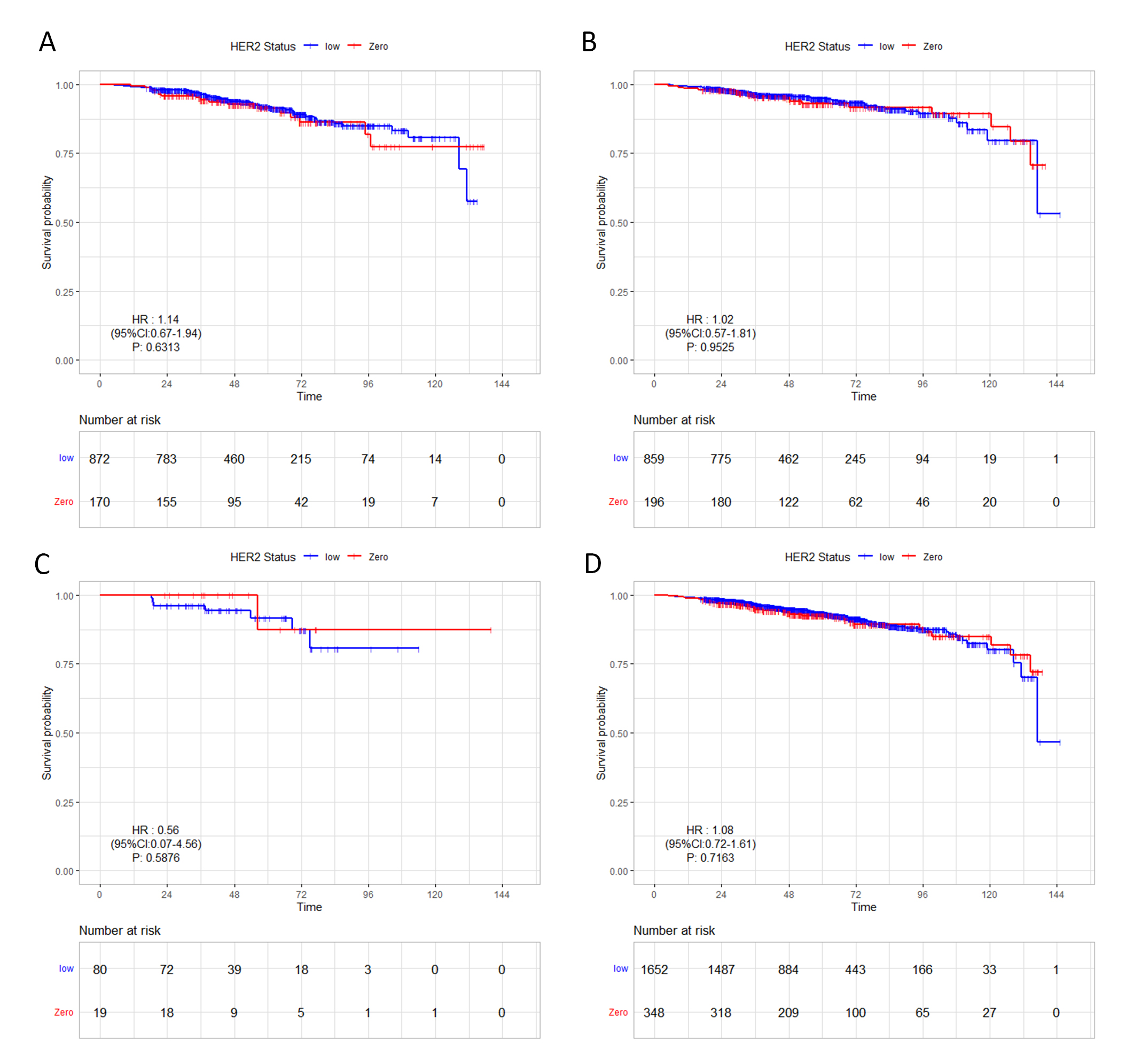

Supplement: Supplementary file 2 — Supplemental Figure 2 Subgroup analysis of DFS in HER2-low and HER2-zero patients. (A) with chemotherapy; (B) without chemotherapy; (C) with OFS; (D) without OFS (JPG 708 kb) [file 12282_2022_1364_MOESM2_ESM.jpg]

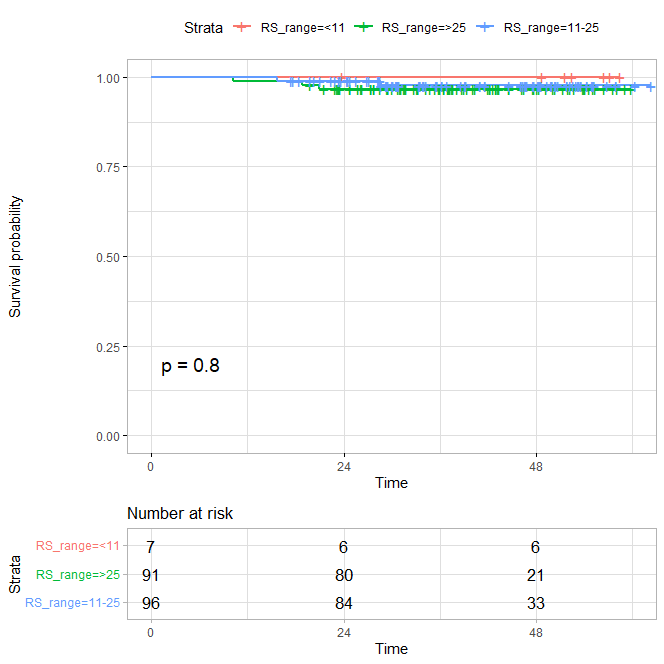

Supplement: Supplementary file 3 — Supplemental Figure 3 DFS of HER2-zero patients diagnosed after Dec 2015 (PNG 18 kb) [file 12282_2022_1364_MOESM3_ESM.png]
