# Supplementary material for: Prognostic values of clinical and molecular features in HER2 low-breast cancer with hormonal receptor overexpression: features of HER2-low breast cancer
Source: Breast Cancer. 2022 Jun 21;29(5):844–53. doi: 10.1007/s12282-022-01364-y (PMC9385837; doi:10.1007/s12282-022-01364-y)
Supplement: Supplementary file 4 — Supplementary file4 (DOCX 21 kb) [file 12282_2022_1364_MOESM4_ESM.docx]

| **Supplemental Table 1 Postoperative chemotherapy in HER2-low and HER2-zero patients with different genetic risks** | | | |
| --- | --- | --- | --- |
| **Treatment** | HER2-zero(%) | HER2-low(%) | p |
| **Low-risk**  **Chemotherapy**  **Yes**  **No** | 12(21.8)  43(78.2) | 49(22.6)  168(77.4) | 0.99 |
| **Median-risk**  **Chemotherapy**  **Yes**  **No** | 58(34.9)  108(65.1)) | 348(40.6)  509(59.4) | 0.20 |
| **High-risk**  **Chemotherapy**  **Yes**  **No** | 100(69.0)  45(31.0) | 475(72.3)  182(27.7) | 0.48 |

Abbreviation: HER2, human epidermal growth factor receptor 2

| Supplemental Table 2 The impact of RS modules and genes on DFS according to HER2 status adjusted by TN stage | | | | |  |
| --- | --- | --- | --- | --- | --- |
| Factors | HER2-negative | p-value | HER2-low | p-value | p_interaction_ |
| ER Module  ER  PgR  Bcl2  SCUBE2  ER Module | 1.17(0.85-1.62)  0.84(0.67-1.06)  0.94(0.62-1.41)  0.90(0.71-1.15)  0.85(0.59-1.22) | 0.33  0.14  0.76  0.40  0.37 | 0.98(0.84-1.15)  0.99(0.89-1.10)  0.90(0.74-1.10)  0.99(0.88-1.10)  0.96(0.79-1.15) | 0.83  0.85  0.31  0.82  0.63 | 0.39  0.21  0.63  0.31  0.39 |
| Proliferation Module | | | | |  |
| Ki67  STK15  Survivin  CCNB1  MYBL2  Prol Module | 2.09(1.38-3.15)  1.70(1.15-2.50)  1.59(1.15-2.20)  1.38(0.84-2.27)  1.52(1.13-2.06)  5.27(1.72-16.17) | <0.01  <0.01  <0.01  0.20  <0.01  <0.01 | 0.98(0.83-1.16)  0.98(0.84-1.15)  1.00(0.84-1.20)  1.16(0.92-1.46)  1.03(0.86-1.23)  0.71(0.35-1.45) | 0.83  0.83  0.98  0.22  0.75  0.57 | <0.01  0.01  0.03  0.64  0.07  <0.01 |
| HER2 Module |  |  |  |  |  |
| GRB7  HER2  HER2 Module | 1.43(1.05-1.95)  1.03(0.71-1.49)  1.41(0.73-2.73) | 0.02  0.91  0.31 | 1.00(0.82-1.24)  0.97(0.79-1.20)  1.96(1.07-3.61) | 0.96  0.79  0.03 | 0.12  0.86  0.32 |
| Invasion Module | | | | | |
| MMP11  CTSL2  Inv Module | 1.41(0.73-2.73)  1.36(1.04-1.78)  1.56(1.02-2.36) | 0.31  0.03  0.04 | 1.96(1.07-3.61)  1.05(0.88-1.25)  1.27(1.03-1.56) | 0.03  0.62  0.03 | 0.53  0.32  0.70 |
| GSTM1 | 1.22(0.87-1.71) | 0.25 | 0.88(0.73-1.06) | 0.17 | 0.31 |
| CD68 | 1.54(1.01-2.37) | 0.05 | 1.08(0.85-1.37) | 0.54 | 0.32 |
| BAG1 | 1.20(0.76-1.88) | 0.43 | 1.03(0.84-1.27) | 0.78 | 0.95 |
